# Augmented reality for guiding resuscitation in rare cardiac channelopathies: a call for innovation in emergency care

**DOI:** 10.1097/MS9.0000000000003761

**Published:** 2025-08-28

**Authors:** Muddassir Khalid, Sarah Omer, Muhammad Asad Rehman, Supoma Gosh Ria

**Affiliations:** a Department of Medicine, Nishtar Medical University, Multan, Pakistan; b Department of Medicine, Quaid-e-Azam Medical College, Bahawalpur, Pakistan; c Mugda Medical College, University of Dhaka, Dhaka, Bangladesh

Augmented Reality (AR) is a type of Virtual Reality innovation that integrates complex medical visuals into the real-world surroundings of a user to improve management during complex procedures. A virtual setting enables the tangible, real-time integration of data from various sources, providing caregivers with a convenient understanding, thus complementing traditional learning methods^[1]^. An undiscovered yet promising area of its application is emergency resuscitation of patients with rare cardiac channelopathies, including Long QT syndrome, Brugada syndrome, and short QT syndrome, where timely and targeted management is critical. According to recent studies, these channelopathies, often underdiagnosed, account for a significant percentage of the Sudden Cardiac Death (SCD) among people under 35 years. In the case of such cardiac emergencies, the traditional resuscitation measures are ineffective or even harmful if not adapted to standard electrophysiology of these channelopathies^[2]^. According to evidence, anti-arrhythmic drugs like procainamide can unmask Brugada syndrome, inducing arrhythmogenesis that leads to ventricular tachycardia. Additionally, amiodarone may exacerbate arrhythmia and affect defibrillation timing due to sodium channel blockade^[3]^. These findings have raised the need to introduce a rapid context-based and targeted approach to address it, where AR can be a good option for guiding catheter ablation and implantable cardioverter defibrillator ICD, which are first line for channelopathies. AR provides real-time, patient-specific 3D visual guidance by integrating ECG interpretation with visual fluoroscopy information, thereby facilitating cardiovascular interventions. AR can serve as a significant interactive cognitive aid, visualizing important cues related to underlying pathology and highlighting the critical deviations from standard ACLS protocols, to reduce human error in high-stress emergency cases like rare channelopathies^[4]^.

The AR technology has exceptional potential for enhancing efficiency, decision-making & improving accuracy in multiple procedures such as trans-catheter aortic valve implantation and percutaneous coronary interventions to electrophysiology mapping and ablation. By providing real-time visuals, clinicians can visualize the patient’s unique cardiac anatomy and the live positioning of interventional devices, enabling extraordinary precision during catheter-based interventions and ablation. In a typical catheterization laboratory setting, augmented reality (AR) can create realistic, interactive 3D digital holograms. These holograms can be utilized in clinical medical imaging^[5]^. Being affordable and portable hardware, its integration into emergency services, trauma bays, and even rural healthcare settings is very feasible and even a need of the hour to reduce the deaths due to rare but fatal channelopathies significantly. AR brings the hope for shifting the strategy of emergency resuscitation from protocol-driven to precision-guided care^[6]^. Despite notable advancements, several obstacles still need to be overcome to increase the quality outcomes of AR technologies in interventional cardiology. It calls for further studies to establish its standardized protocols, cost-effectiveness, potential adverse effects, and impact on actual resuscitation performance in real-life scenarios to advocate its incorporation in emergency measures.

TITAN Guidelines: This manuscript is in compliant to TITAN Guidelines, 2025, declaring no use of AI^[7]^.

## Data Availability

Not applicable.
